# Invasive colon cancer, but not non-invasive adenomas induce a gradient effect of Wnt pathway receptor frizzled 1 (Fz1) expression in the tumor microenvironment

**DOI:** 10.1186/1479-5876-11-50

**Published:** 2013-02-26

**Authors:** Kestutis Planutis, Marina Planutiene, Anthony V Nguyen, Mary Pat Moyer, Randall F Holcombe

**Affiliations:** 1 Division of Hematology/Oncology, Tisch Cancer Institute, Mount Sinai School of Medicine, New York, NY, USA; 2 Division of Hematology/Oncology, University of California, Irvine, Irvine, California, USA; 3 INCELL Corporation, San Antonio, Tx, USA

**Keywords:** Wnt signaling, Colon cancer, Tumor microenvironment, Fz receptors, Wnt3a

## Abstract

**Background:**

Wnt signaling in the colon cancer tumor microenvironment (TME) may affect cancer biologic properties including invasion and metastatic dissemination. Prior reports have suggested that the expression of select frizzled (Fz) receptors may be altered in cancers and in the TME.

**Methods:**

Colon cancer, colonic adenoma and normal colonic mucosal specimens were obtained under institutional review board approval and analyzed for the expression of Fz1 and Fz2 by confocal fluorescent immunohistochemistry and Wnt-specific membrane array. In vitro, the effect of Wnt3a on Fz1 expression was examined in normal-derived NCM460 cells by qRT-PCR and immunohistochemistry.

**Results:**

Fz1 was expressed in colon cancer and villous adenomas but not in more benign tubular adenomas. Fz1 expression was seen in normal colonic mucosa in close proximity to colon cancer, but not villous or tubular adenomas. Normal colonic mucosa distant from colon cancer did not express Fz1. Fz2 was expressed ubiquitously in cancer, adenomas and normal colonic mucosa. Fz1 expression was induced by Wnt3a in a normal colon mucosa-derived cell line in vitro.

**Conclusions:**

Fz1 is a Wnt responsive gene in colon-derived tissues. Fz1 expression exhibited increased expression in normal mucosa only in close proximity to colon cancer. This field effect was not seen with pre-malignant adenomas and may be due to Wnt/β-catenin signaling within the TME. Fz1 may represent a new TME-directed therapeutic target for patients with colon cancer.

## Background

There is increasing recognition that tumor biology is modulated by signals derived from, and received by cells within the tumor microenvironment (TME) [1]. Interactions with non-transformed cells in the TME may facilitate invasion and metastases, or, alternatively, inhibit tumor progression. Enhanced Wnt pathway signaling has been recognized as a hallmark of colon cancer with over 85% of these malignancies harboring mutations within the pathway that lead to constitutive activation [2]. However, the strength of the Wnt signal is modulated by factors within the TME, including Wnt ligands which augment canonical Wnt signaling and soluble inhibitors which suppress it [3,4].

Wnt activity in colon cancer stem cells is modulated by myofibroblast-secreted factors in the TME [5] and other stromal cells may similarly regulate Wnt activity in colon cancer in a paracrine fashion [6]. Interactions of malignant with stromal cells may affect Wnt signaling directly [7] or via autocrine feedback loops [8] from tumor cells overexpressing secreted Wnt ligands [9]. The invasion margin of colon cancer is characterized by nuclear b-catinin suggesting increased Wnt pathway activation at the cancer:stroma interface [10]. While most studies have focused on the effects of stroma on the biologic behavior of the cancer cells, Wnt signaling in the TME can also directly affect the tumor stroma, with subsequent secondary effects on cancer invasion and progression [11].

Frizzled receptors are 7-transmembrane proteins which serve, in conjunction with LRP5/6 co-receptors as the binding partners for extracellular Wnt ligands to initiate the Wnt signal. Conceptually, the signals transduced progress along one of two intracellular pathways – canonical, resulting ultimately in the accumulation of β-catenin in the nucleus where it promotes transcriptional activation in conjunction with the LEF/TCF family of DNA binding proteins and non-canonical, resulting in activation of protein kinase C, c-Jun-N-terminal kinases or calcium/calmodulin-dependent kinases. However, Wnt signaling is significantly more complex as there is crosstalk between canonical and non-canonical pathways, and ligands and receptors may result in differential signaling in a context-dependent fashion [12].

Few studies have examined the role of distinct frizzled receptors in the TME. Factors in the TME have been implicated in the regulation of Fz7 on colorectal cancer cells [13] and Fz7 downregulation has been linked to reduced survival, invasion and metastatic potential [14], suggesting that Fz7 may represent a therapeutic target. In this report, we describe the differential expression of Fz1 in the colon cancer TME and provide evidence of autocrine regulation in tumor cells and paracrine regulation by factors within the TME on Fz1 expression in non-malignant mucosal epithelium, as well as lack of paracrine regulation in proximity to pre-malignant colon adenomas. To our knowledge, this is the first report of a field effect of a specific frizzled receptor in the colon tumor microenvironment.

## Materials and methods

### Tissue acquisition

Over 20 distinct de-identified colon cancer and corresponding normal colonic mucosa was obtained from archived specimens at UCIrvine under a non-human subjects exemption by the UCIrvine institutional review board. Adenoma specimens with biopsies of corresponding normal mucosa were obtained from 12 different patients following informed consent under a UCIrvine institutional review board approved protocol from patients undergoing elective colonoscopic screening as described previously [15]. All research was performed in compliance with the Declaration of Helsinki.

### Cell culture

The NCM460 cell line was purchased from INCELL Corporation (San Antonio, TX, USA) and cultured in M3 media supplemented with 10% FBS, 100 U/ml penicillin and 100 mg/ml streptomycin at 37°C in a 95% humidified atmosphere with 5% CO2. This epithelial cell line was derived from normal colonic mucosa and is not tumorigenic though, over time in culture it has acquired some transformation-associated characteristics [16]. NCM460 is not transfected with any exogenous genetic information.

### WNT3a conditioned medium (CM)

Wnt3a CM and control CM were prepared using the LWnt3A cell line (ATCC#CRL-2647) and the parental cell line (ATCC #CRL-2648) according to procedures perfected in the laboratory of Dr. Roel Nusse [17]; for a preparation protocol, see http://www.stanford.edu/group/nusselab/cgi-bin/wnt/. Cells were analyzed after 24 h of Wnt3a treatment for Fz1 expression.

### Immunohistochemistry

Immunohistochemistry for Fz1 and Fz2 were performed with specific anti-Fz1 antibodies (Genentech, Inc., #GTX71477) and anti-Fz2 antibodies, (Genentech, Inc. #GTX71817), 1 mg/ml, at 1:200 dilution. Paraffin-imbedded slides were deparaffinized and sodium citrate buffer pH 6 20 min at 99°C was used as an antigen retrieval agent. Santa Cruz Biotechnology rabbit ABC Staining System (SC-2018) was used according to the manufacturer’s protocol, and the signal was amplified by TSA™ Fluorescein Tyramide Reagent from Perkin Elmer (SAT701001EA). Slides were stained with DAPI and covered with anti-fade embedding medium (ProLong Gold antifade reagent, Invitrogen P36934). Confocal microscopy was performed with a Carl Zeiss LSM 510 and 40x fluorescent objective.

### Quantitative real-time polymerase chain reaction (qRT-PCR)

Fz1 expression in NCM460 was determined by qRT-PCR with Fz1 specific primers. The efficiency of the Fz1 and actin primers was equivalent. Primer pairs were obtained from Qiagen (Valencia, CA) with cycling parameters as defined by the manufacturer. For qRT-PCR experiments, the relative RNA expression was calculated by the comparative threshold cycle method.

### Wnt-specific membrane array

Fz1 mRNA expression in adenomas and normal mucosa was defined with cDNA generated from tissue-derived RNA and analyzed with a GEArray Q Series Human Wnt Signaling Pathway Gene Array (SuperArray Bioscience, Frederick, MD). GEArray expression analysis software was utilized for background normalization, correction for different degrees of exposure, and normalization with multiple housekeeping gene controls on each membrane.

### Statistical analysis

Fz1 mRNA and protein expression in NCM460 cells was compared with an unpaired *t*-test between experimental (Wnt3a CM) and control (l-Cell CM) groups. Expression of Fz1 in adenomas, was analyzed across the 3 groups by 1-way ANOVA with a post-hoc Newman Kuels test. Individual subgroups were also compared by unpaired *t*-test.

## Results

### Expression of Fz1 and Fz2 receptors in colon cancer tumor microenvironment

**Figure 1:**
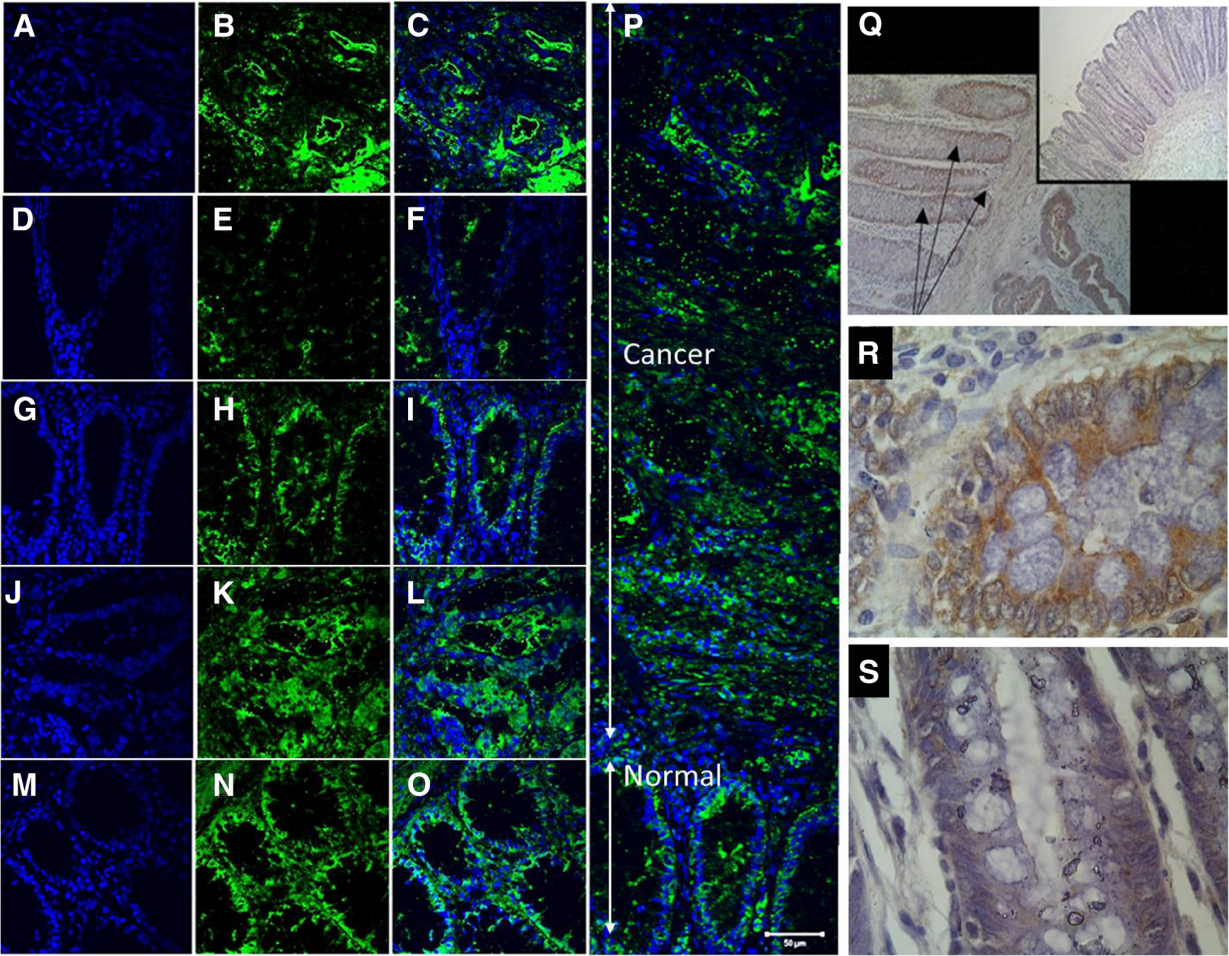
**Expression of frizzled receptors by fluorescent immunohistochemistry in patient derived samples.** Panels **A**, **B**, **C**: Fz1 expression in colon cancer – DAPI (**A**), anti-Fz1 antibody (**B**), merged images (**C**) – showing uniform expression. Panels **D**, **E**, **F**: Fz1 expression in normal colonic mucosa distant (>2 cm) from colon cancer – DAPI (**D**), anti-Fz1 antibody (**E**), merged images (**F**) – showing lack of significant expression. Panels **G**, **H**, **I**: Fz1 expression in normal colonic mucosa adjacent to colon cancer – DAPI (**G**), anti-Fz1 antibody (**H**), merged images (**I**) – showing significant Fz1 expression. Panels **J**, **K**, **L**: Fz2 expression in colon cancer – DAPI (**J**), anti-Fz2 antibody (**K**), merged images (**L**) – showing uniform expression. Panels **M**, **N**, **O**: Fz2 expression in normal colonic mucosa distant (>2 cm) from colon cancer – DAPI (**M**), anti-Fz2 antibody (**N**), merged images (**O**) – showing uniform expression. Panel **P**: Composite of multiple merged images of Fz1 expression in colon cancer and normal colonic mucosa adjacent to tumor showing expression in both the cancer and in the adjacent normal mucosa. Scale bar = 50 um. Panel **Q**: DAB immunohistochemistry with anti-Fz1/(2) antibody of colon cancer, normal colonic mucosa adjacent to the tumor (arrows) and normal colonic mucosa distant from the tumor (insert) showing significant expression in the cancer and in the adjacent normal colonic mucosa. Panel **R**: Higher magnification from image displayed in Panel **Q** of normal colonic mucosa adjacent to colon cancer showing expression. Panel **S**: Higher magnification from image displayed in Panel **Q** of normal colonic mucosa distant from colon cancer showing lack of expression.

Fluorescent immunohistochemistry with specific anti-Fz1 and anti-Fz2 antibodies was performed on tissue sections from colon cancer, adjacent normal colonic mucosa and distant normal colonic mucosa. All experiments included secondary antibody only controls on sequential tissue sections. Fz1 and Fz2 were both expressed in colon cancer tissues. Fz1 was detected in histologically normal mucosa in close proximity to the neoplastic cells (see Figure 1, panels H and P). The normal mucosa examined was adjacent to an invasive margin of the tumor. More distant from the tumor, however, Fz1 expression was not seen in normal mucosa demonstrating a field effect in the tumor microenvironment with respect to expression of this specific Wnt receptor. In contrast, Fz2 was expressed ubiquitously in normal colonic mucosa in addition to its expression in colon cancer.

### Induction of Fz1 expression in vitro

**Figure 2:**
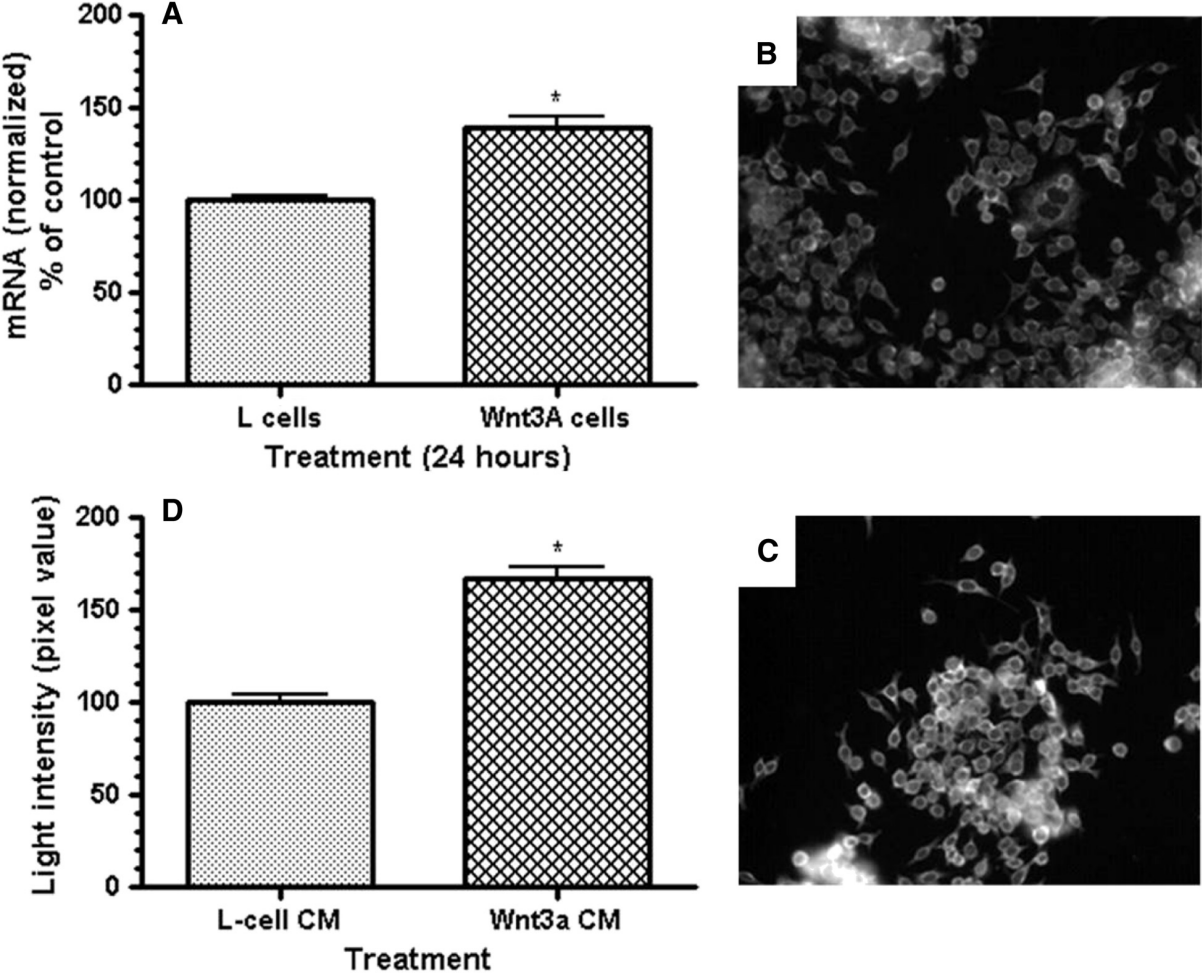
**Response of Fz1 expression to exogenous Wnt3a ligand in normal colon-derived NCM460 cells.** Panel **A**: mRNA levels by quantitative real-time PCR without and with 24 h of exposure to Wnt3a. p<0.005 for the difference (3 separate experiments, all in triplicate). Panels **B**, **C**: Fluorescent immunohistochemistry of NCM460 cells in tissue culture without (**B**) and with (**C**) 24 h of exposure to Wnt3a. Panel **D**: Quantified light intensity from immunofluorescence of anti-Fz1 antibody staining in NCM460 cells without and with 24 h of exposure to Wnt3a. p<0.001 for the difference (3 independent experiments with 25 individual fields recorded for each).

In tissue culture, normal colon-derived NCM460 cells were evaluated for expression of cell surface Fz1 in response to exogenous Wnt ligand Wnt3a. These experiments demonstrate that Fz1 is a Wnt-responsive gene, with increased expression in response to Wnt3a. The Wnt3a response was manifested by both an increase in Fz1 mRNA (p<0.005; Figure 2, panel A) and Fz1 protein (light intensity, p<0.001; Figure 2, panel D), suggesting that enhanced transcription is the primary mechanism leading to increased Fz1 expression in response to Wnt3a.

### Expression of Fz1 in colonic adenomas

**Figure 3:**
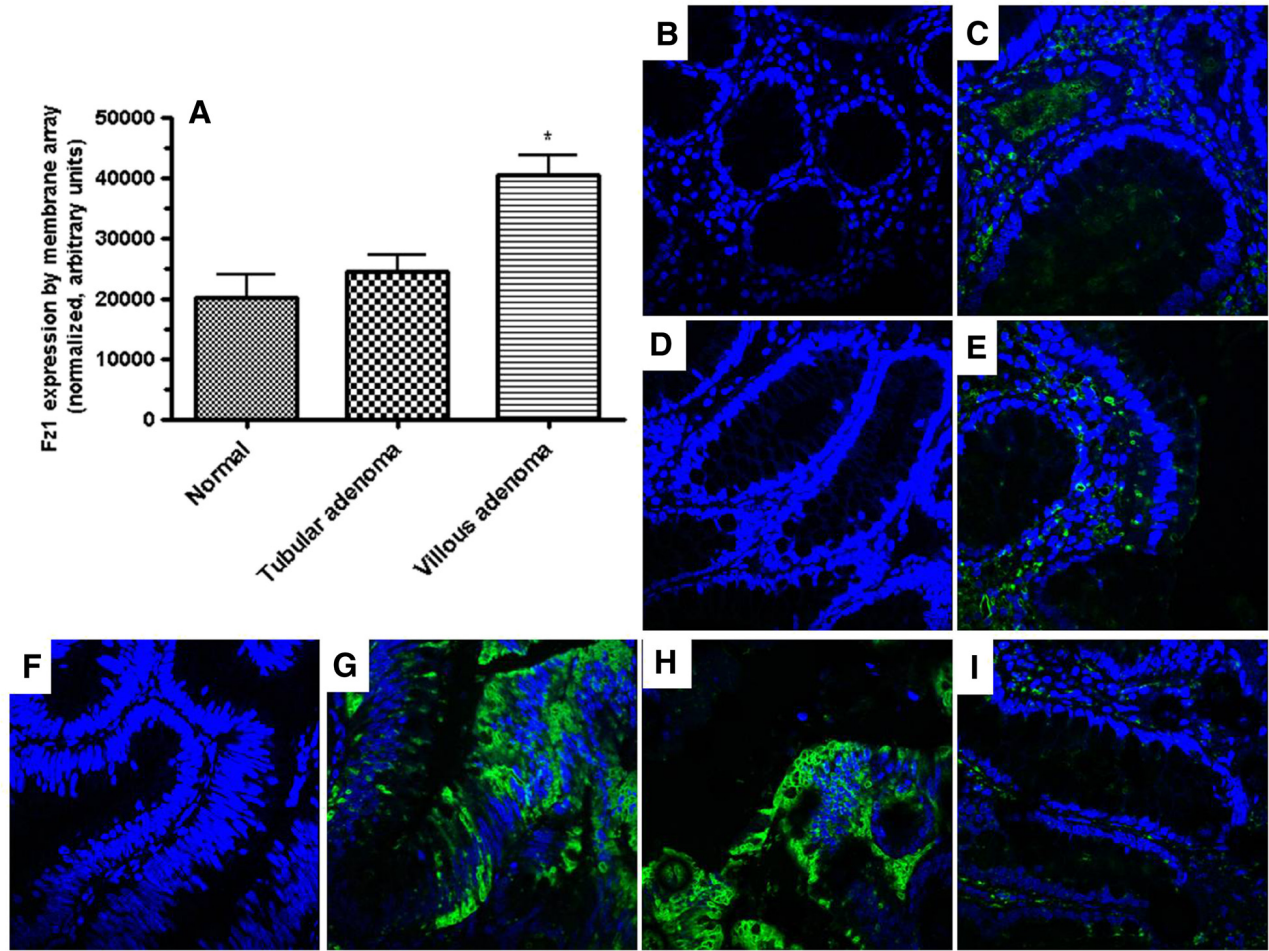
**Expression of Fz1 in colonic adenomas.** Panel **A**: mRNA levels by Wnt specific membrane array in normal colonic mucosa, tubular adenoma and villous adenomas (N=12). p=0.0176 (1 way ANOVA) with p<0.05 by Newman-Kuels post hoc analysis for villous vs. normal and villous vs. tubular; p=0.024 villous vs. tubular (unpaired Student’s *t* test); p=0.012 villous vs. normal (unpaired Student’s *t* test). Panel **B**, **C**: Normal colonic mucosa – secondary antibody only (**B**) and merged images (**C**) - showing lack of Fz1 expression. Both panels co-stained with DAPI. Panel **D**, **E**: Tubular adenoma – secondary antibody only (**D**) and merged images (**E**) - showing lack of Fz1 expression. Both panels co-stained with DAPI. Panel **F**, **G**, **H**: Villous adenomas – secondary antibody only (**F**) and 2 merged images (**G**, **H**) of villous adenomas from different patients. Staining with anti-Fz1 (**G**, **H**) antibody demonstratesFz1 expression. All panels co-stained with DAPI. Panel **I**: Merged image of normal colonic mucosa adjacent to villous adenoma showing lack of Fz1 expression.

Colonic adenomas were evaluated for Fz1 expression in order to define the stage in the neoplastic progression where Fz1 expression begins and whether the field effect for Fz1 noted in normal mucosa adjacent to colon cancer is specific for invasive carcinoma. Using a Wnt specific membrane array, Fz1 mRNA could be detected at low levels in both normal colonic mucosa and tubular adenomas. However, Fz1 mRNA was markedly increased in villous adenomas in comparison to normal mucosa and tubular adenomas (p=0.0176 1 way ANOVA, p<0.05 Newman-Kuels for both comparisons; p=0.024 villous vs. tubular by unpaired *t*-test; p=0.012 villous vs. normal by unpaired *t*-test). Villous adenomas have a substantially higher malignant potential than tubular adenomas.At the protein level, villous adenomas had abundant Fz1 expression (Figure 3, panels G and H) but no expression was detected in normal mucosa or tubular adenomas, suggesting that the molecular switch or conditions within the tumor microenvironment permissive for Fz1 expression occur in the transition from tubular to villous histologies.. In contrast to what was seen with invasive colon cancer, however, no field effect of the expression of Fz1 was seen in close proximity to non-invasive adenomas (Figure 3, panel I), including villous adenomas which themselves expressed Fz1 protein.

## Discussion/conclusions

The expression of the Wnt pathway receptors Fz1 and Fz2 has been previously implicated in cancer development and progression. Their expression is reported to be increased in breast cancer in comparison to normal breast epithelium [18]. There is also prior evidence using older, less specific antibodies that Fz1 or Fz2 expression is increased at the invasion margin of poorly differentiated colon cancer [9]. Fz1 mediates Wnt/β-catinin (canonical) signaling in neuroblastoma [19] and serves as a receptor for Wnt3a in both macrophages [20] and pheochromocytoma-derived PC12 cells [21]. In both these latter systems, Wnt3a/Fz1 interactions induce signaling along the canonical Wnt pathway. However, there is one report that Fz1 is antagonistic of Wnt/β-catinin signaling in a Xenopus cap assay and secondary axis formation assay [22], suggesting that the specific activity of Fz1 may be context dependent. The role of Fz2 is less well studied though it appears to bind to both Wnt3a, which promotes Wnt/β-catinin signaling and Wnt5a which inhibits it [23].

What is clear from this report is that Fz1 expression is tightly regulated in the tumor microenvironment. As both an effector molecule for Wnt signaling and also, as we have shown here, as a Wnt responsive gene, Fz1 may function as part of a positive autocrine feedback loop in colon cancer cells. Whether this effect is limited to Wnt3a which promotes Fz1 transcription and to which Fz1 also binds requires further study. In the tumor microenvironment, a field effect of Fz1 expression exists suggesting paracrine regulation of this receptor. Fz1 expression on normal colonic mucosa within the TME is induced by cancer but not by non-invasive adenomas, including villous adenomas which themselves express Fz1. We speculate that this is related to the degree of Wnt, and particularly Wnt3a ligand production by the colon cancer cells. Increased nuclear localization of β-catenin at the invasion front is a characteristic of colon cancer [24] and colon cancer is known to have increased expression of various Wnt ligands [9]. While this prior work demonstrated differences in Wnt ligand expression between tumor and normal tissues, whether a gradient of Wnt3A expression within the tumors exists, possibly increased at the invasion margin, has not been investigated. Differences in the level of Wnt activity within tumors may occur, with a high activity of the Wnt pathway is observed in tumour cells located close to stromal myofibroblasts, implicating signals from the stroma may be involved [5]. Secreted factors other than Wnt ligands may also be important. For example, Suzuki and colleagues [25] have reported that epigenetic inactivation of SFRP genes leads to augmented Wnt signaling in colon cancer and this process may not be operative in pre-malignant adenomas. Lack of nuclear translocation of β-catenin in sporadic adenomas, in contract to cancer, has been described previously [26]. Finally, expression of Fz1, as well as Fz6 and Fz7, are positively correlated with Wnt/β-catenin signaling in human mesenchymal cells [27] further suggesting that the regulated expression of Fz1 in the tumor microenvironment is a function of, and contributes to, Wnt signaling in the tumor:stromal mileau. While no morphologic change in the composition of stroma was identified in our study, experiments were not designed to specifically detect this. Stromal cell recruitment at tumor sites has been described previously [28]. Further investigations will be needed to define whether Fz1 and Wnt signaling play a role in this process.

In addition to its expression in colon cancer, Fz1 expression appears to be confined to pre-malignant adenomas with villous features which represent the histologic type of adenoma with greatest risk for malignant transformation [29]. Development of rapid immunofluorescence techniques that can be performed in conjunction with advanced endoscopic imaging could potentially lead to the use of Fz1 to differentiate villous from non-villous adenomas in situ. This marker may also prove a useful adjunct to routine histologic examination of colonic adenomas.

The significance of Fz1 on normal colonic mucosa in the TME requires further study. Given the known functions of Wnt/β-catinin signaling, it may promote proliferation and block differentiation of colonic mucosal cells, promoting tumor invasion by accelerating mucosal turnover. Fz1 expression in the TME may modulate colon cancer progression and dissemination and therefore may represent a new TME-directed therapeutic target for patients with colon cancer.

## Competing interests

KP, MP, AVN and RFH have no conflicts of interest to disclose. MPM is owner of INCELL Corporation which markets the NCM460 cell line utilized in this study.

## Authors’ contributions

KP and MP conducted the majority of studies outlined in this study. AVN conducted membrane array studies related to adenomas. MPM provided the cell line for in vitro studies and reviewed the data and manuscript. RFH designed the studies and provided oversight for the project overall. All authors approved the final manuscript.
